# An Oligosaccharide Rich Diet Increases *Akkermansia* spp. Bacteria in the Equine Microbiota

**DOI:** 10.3389/fmicb.2021.666039

**Published:** 2021-05-21

**Authors:** Frederikke Christine Lindenberg, Ditte Olsen Lützhøft, Lukasz Krych, James Fielden, Witold Kot, Hanne Frøkiær, Gaby van Galen, Dennis Sandris Nielsen, Axel Kornerup Hansen

**Affiliations:** ^1^Brogaarden ApS, Lynge, Denmark; ^2^Department of Veterinary and Animal Sciences, Faculty of Health and Medical Sciences, University of Copenhagen, Copenhagen, Denmark; ^3^Department of Food Sciences, Faculty of Sciences, University of Copenhagen, Copenhagen, Denmark; ^4^Department of Environmental Sciences, Aarhus University, Aarhus, Denmark; ^5^Department of Veterinary Clinical Sciences, Faculty of Health and Medical Sciences, University of Copenhagen, Copenhagen, Denmark

**Keywords:** regulatory immunity, gut microbiota, oligosaccharides, prebiotics, *Akkermansia* municiphila

## Abstract

Some oligosaccharides induce growth of anti-inflammatory bacterial species and induce regulatory immunity in humans as well as animals. We have shown that the equine gut microbiota and the immune-microbial homeostasis largely stabilize within the first 50 days of life. Furthermore, we have previously established that certain bacterial species in the equine gut correlated with regulatory immunity. Accordingly, we hypothesized that an oligosaccharide rich diet fed to foals during the first 50 days would increase the abundance of bacterial species associated with regulatory immunity, and that this would influence immune responses in the foals. Eight pregnant mares and their foals were fed an oligosaccharide rich diet from 4 weeks before expected parturition until 49 days post-partum. Six mares and foals served as control. Fecal microbiota from mares and foals was characterized using 16S rRNA gene amplicon high throughput sequencing. On Day 49 the test foals had significantly higher abundances of *Akkermansia spp.* Blood sampled from the foals in the test group on Day 7, 28, and 49 showed non-significant increases in IgA, and decreases in IgG on Day 49. In BALB/cBomTac mice inoculated with gut microbiota from test and control foals we found increased species richness, increased relative abundance of several species identified as potentially anti-inflammatory in horses, which were unclassified Clostridiales, Ruminococcaceae, *Ruminococcus*, *Oscilospira*, and *Coprococcus*. We also found increased *il10* expression in the ileum if inoculated with test foal microbiota. We conclude that an oligosaccharide diet fed to foals in the “window of opportunity,” the first 50 days of life, increases the abundance of anti-inflammatory species in the microbiota with potentially anti-inflammatory effects on regulatory immunity.

## Introduction

Gut microbiota composition and function have a vast influence on the developing immune system (Hansen et al., 2012b, 2013b; Weng and Walker, 2013). The immunological homeostasis is established right after birth in parallel with the initial colonization of the gastrointestinal tract by microorganisms from the birth canal, milk, mother, and the surrounding environment (Dominguez-Bello et al., 2010; Makino et al., 2013; Pannaraj et al., 2017). Oral tolerance toward commensal bacteria and food antigens is mediated through regulatory T-cells (Tregs) and their anti-inflammatory signals such as transforming growth factor-beta (TGF-β) and interleukin 10 (Il-10) (Chen et al., 2003; Round and Mazmanian, 2010; Geuking et al., 2011). Failure to establish a proper gut microbiota may lead to the skewing of the immune-microbial homeostasis such as suggested by Strachan (1989) and also observed in babies born by cesarean section (Dominguez-Bello et al., 2010; Hansen C. H. F. A. et al., 2014). This results in an imbalance between the effector and regulatory T-cells favoring a more reactive T-cell phenotype and increased risk of inflammatory disorders (Cani et al., 2008; Hansen et al., 2012b; Hansen A. K. et al., 2014). Colonization by the same bacteria before or after establishment of the immune-microbial homeostasis yields very different immunological reactions (Hansen et al., 2012b). Consequently, early life gut microbiome development may have long-lasting immunological effects beyond those of the immediate stimulation, and therefore it is possible to permanently influence the immune system through gut microbiota manipulation (Hansen et al., 2012b; El Aidy et al., 2013).

We have previously shown that especially members of the Clostridiales order and Verrucomicrobia phylum correlate positively with increased expression of the Forkhead box P3 (Foxp3) transcription factor, TGF-β, and IL-10, all of which are markers of Treg activity (Lindenberg et al., 2019a) indicating that the gut microbiota in horses can modulate immunity. In humans and mice both the Clostridiales spp. *Faecalibacterium prausnitzii* and the Verrucomicrobia spp. *Akkermansia muciniphila* are known to be very potent inducers of regulatory immunity and have been linked to prevention or amelioration of several inflammatory disorders (Atarashi et al., 2011; Hansen et al., 2012a; Qiu et al., 2013; Shin et al., 2014). The oligosachharides galacto- and fructo-oligosaccharides (GOS and FOS) have been shown to increase the abundance of Clostridiales spp. in the gut of newly weaned infants (Scholtens et al., 2006). Additionally, in a mouse model of human obesity and diabetes, mice fed fructooligosaccharides (FOS) had increased proportion of *A. muciniphila* and reduced obesity and inflammation (Everard et al., 2013). It is, therefore, reasonable to assume that horses would be able to increase their regulatory immunity, through oligosaccharide feeding increasing the presence of members of Clostridiales and Verrucomicrobia.

In recent years, various oligosaccharides have been used as pre-biotic food supplements, for both humans and animals, to promote the growth of anti-inflammatory bacteria species in the gut microbiota. Their therapeutic applications in relation to metabolic and inflammatory diseases such as type 2 diabetes (T2D) and obesity have been shown in mice and rats (Gobinath et al., 2010; Hansen et al., 2013a), and humans (Parnell and Reimer, 2009). Mannanoligosaccharides (MOS) has been shown to increase the colostrum levels of the immunoglobulins (Ig) IgA and IgG in mares (Spearman and Ott, 2004) and act as alternative binding sites for gastrointestinal pathogens in chickens (Spring et al., 2000; Baurhoo et al., 2007). High doses FOS have traditionally been used to induce a model of hindgut acidosis and laminitis in horses (van Eps and Pollitt, 2006; Bailey et al., 2009; Moreau et al., 2014). Recent studies showed that low doses of FOS supplementation had a stabilizing effect on the equine microbiota during sudden diet change and significantly decreased the basal plasma insulin level in insulin resistant horses (Respondek et al., 2008, 2011), suggesting a beneficial effect of low doses of FOS. It has been shown that neonatal IgA production in mice is regulated by the maternal microbiota (Mu et al., 2021).

In spite of the convincing evidence from other species, very little research has been conducted into the effects of prebiotics on the development of the gut microbiota and the immune system in horses. The aim of this study was to determine whether it was possible to stimulate the development and colonization of a gut microbiota by feeding a mixture of potentially beneficial oligosaccharides to mares and foals. This would in turn promote regulatory immunity in newborn foals. We hypothesized the following: a mixture of MOS and FOS will promote the growth of anti-inflammatory bacteria in the mare GIT which will be passed on to colonize the foal. This will affect the immune responses as assessed by the production of antibodies in the foal serum and by a mouse model of the local immune-microbial interaction in the horse gut. Finally, there will be an additive and more direct effect when the foals start eating the oligosaccharides themselves. Of particular interest was the effect of this diet regiment on the abundance of bacteria that we have previously correlated to regulatory immunity.

## Materials and Methods

Two animal studies were conducted. A 78 days intervention study in pregnant mares and foals, and a 5 week inoculation study in mice.

### Equine Study

#### Ethics Statement

All assays were carried out on samples collected as part of the stud farm’s routine health surveillance, by an authorized veterinarian, which according to the Animal Experimentation Act (LBK No. 474 15/05/2014), should not be regarded as animal experimentations. All procedures were carried out according to The Danish Veterinary Act (Dyrlægeloven) no 48 of 11/01/2017. Approval was obtained from the owners prior to the study start.

#### Animals and Collection of Samples

Fourteen mare and foal pairs were included in the study. All horses were thoroughbred and housed at a stud farm in Hørsholm, Denmark under standard hygienic conditions. Prior to parturition the mares were randomly assigned to the test (eight pregnant mares) or control group (six pregnant mares) and paired according to expected parturition date. The foals were born between January and May 2016. Gestation lengths varied from 331 to 352 days. Two mares (one from each group) needed veterinary assistance during parturition and received post-partum antibiotics (one dose penicillin/gentamycin intra muscularly). Serum IgG were measured 12 h post-partum on a semi-quantitative enzyme-linked immunosorbent assay (ELISA) quick-test (KRUUSE IgG Foal Quick Test, KRUUSE, Langeskov, Denmark). Foals with an IgG level below 400 mg/dl received colostrum replacement paste (FoalGard, Hunden & Herden, Sjöbo, Sweden) 12 h post-partum. In total three foals had IgG levels lower than 400 mg/dl. One foal (control group) was from a mare, which experienced a difficult labor and did not receive colostrum until 6 h after birth. Another one (test group) was from a mare that had been leaking colostrum several days prior to parturition. The third foal (control group) had no obvious reason that could explain the lower level. All other foals had IgG at or above 800 mg/dl. No other replacement in the form of bottle- or tube feeding or intravenous plasma was given. Ten of the foals developed mild self-limiting “foal heat diarrhea” around the age of 2 weeks of life (five from the test group and five from the control group), but none showed any signs of being systemically affected by this. Mares were housed with their foals in individual boxes (one box per mare/foal pair) and let out in a pen separately from other pairs for the first month after giving birth. New straw was added to the boxes daily and boxes were mucked out once a week.

#### Diet

The mares were fed a mixture breeding diet (1–1.5 kg/day) (Optimal nr. 1 Suregrow, Brogaarden Diets Ltd., Lynge, Denmark) as well as alfalfa (0.75–1 kg/day) (Brogaarden Lucerne Mix., Brogaarden Diets Ltd., Lynge, Denmark) (Supplementary Table 1) and hay ad libitum. No nutritional analysis of the hay was available, but all hay fed during the experimental period was from the same batch. Post-partum the mixture breeding diet was increased from 180 g/100 kg BW to 300 g/100 kg BW. The foals were not fed separately but had access to the mares’ food at all times. From 1 month post-partum daily feed rations were gradually increased to 1.5–2.3 kg/day as foals started eating more of the mares’ food. The test group received a supplement in a proprietary blend of oligosaccharides (Brogaarden Diets Ltd., Lynge, Denmark) MOS (21.6%), FOS/Inulin (66.4%) and yeast byproduct/beta-glucans, anti-clogging agent and taste enhance. The mares received 25 g supplement as a top dressing twice daily, which amounts to 0.1 g/kg BW/day for 4 weeks prior to parturition and for 2 months after, this as the end point. The dose of the test supplement was decided upon by assessment of doses used in studies with the aim to induce hindgut acidosis and equine studies using FOS as a prebiotic. Studies inducing hindgut acidosis and laminitis used between 5 and 12.5 g FOS/kg BW and studies using FOS as prebiotic used between 0.01 and 0.08 g FOS/kg BW (see Supplementary Table 2). The mares were monitored for clinical signs such as colic, diarrhea and laminitis throughout the study. As we and others have previously shown that around Day 50 post-partum, the majority of the equine gut microbiota members seems to have reached a stable abundance (Earing et al., 2012; Lindenberg et al., 2019b) we chose this time as end point for the study.

#### Fecal Sample Analysis

Parturition was considered as Day 0. Fecal samples were collected from mares at Day −7, 1, 7, and 49. At Day 0 sampling of foal fecal matter commenced, starting with the first sample collected within 24 h post parturition or as soon as the first fecal matter that was not meconium was seen (Day 1), at day 7 and ended at Day 49. Fecal samples were collected from the ground immediately after dropping from the rectum. The first samples were collected by holding a collection tube directly under the rear of the foal while defecating as the first feces is very watery and not possible to collect from the floor. A subsample of 100–200 g (or as much as possible in very young foals) of fecal matter that had not been in contact with the ground, was collected into a sterile 50 mL Falcon Tube (Thermo Fisher Scientific, Denmark) and stored at −20°C in a mobile freezer for maximum 3 h until it could be stored permanently at −80°C. pH was measured using an on-site pH meter (B-712 LAQUAtwin Compact pH meter. Horiba, Triolab, Brøndby, Denmark) immediately in a 5 g subsample of the extracted fecal matter from the mares to ensure that the test supplement did not cause a significant decrease in pH, as pH is an indicator of the gastro-intestinal health.

Sequencing analysis was performed on paired mare-foal samples taken at Day −7 (only mares), Day 1, Day 7, and Day 49. DNA extraction was performed using the MoBio Power Soil Kit (QIAGEN Nordic, Copenhagen). A pre-step was added to extract the hay and straw from the samples. More specifically, 40 g of thawed fecal sample was suspended in 80 mL sterile milli-Q water in a filter bag (Stomacher 400 classic strainer sterile lab blender bag, Seward Ltd., West Sussex, United Kingdom) and processed in a stomacher (Seward 80 BA 7020 Stomacher Lab Blender, Seward Ltd., West Sussex, United Kingdom) for 2 min at maximum speed, 1.5 mL of fecal slurry was transferred to Eppendorf tubes and centrifuged at 15 × *g* for 10 min. The pellet was transferred to the power bead tubes and the DNA was extracted according to the manual.

#### High Throughput Sequencing of the Gut Microbiota

The fecal microbiota profiles of horses were determined using tag-encoded 16S rRNA gene NextSeq-based (Illumina, San Diego, CA, United States) high throughput sequencing, amplifying the V3 region. The sequencing library preparation steps were conducted as previously described (Williams et al., 2017).

The raw dataset containing pair-ended reads with corresponding quality scores were merged and trimmed using settings as previously mentioned (Williams et al., 2017). Quantitative Insight Into Microbial Ecology (QIIME) open source software package (1.7.0 and 1.8.0) (Caporaso et al., 2010) was used for subsequent analysis steps. Purging the dataset from chimeric reads and constructing de novo operational taxonomic units (OTUs) was conducted using the UPARSE pipeline (Edgar, 2013). The green genes (13.8) 16S rRNA gene collection was used as a reference database (McDonald et al., 2012).

#### Blood Sampling Analysis

Blood was collected as a part of veterinary health monitoring from the foals when they were 7, 28, and 49 days old. Blood was drawn from the jugular vein by vacutainer needle into 5 mL uncoated vacuum tubes (BD, Kruuse, Langeskov, Denmark) and allowed to clot. The samples were centrifuged at 1,000 × *g* for 10 min serum was extracted (1–2 mL), and frozen at −80°C until processing.

#### Analysis of IgG and IgA

Serum samples were analyzed by ELISA for the level of IgA and IgG to assess the immunological effect of the establishment of the microbiota, and potential differences caused by the test supplement. Serum was thawed and a direct ELISA with equine specific monoclonal antibodies for IgG and IgA was performed using the Abcam ELISA kits (Abcam, 330 Cambridge Science Park, Cambridge, United Kingdom). Serum was added to microtiter wells with absorbed antibodies on the surface. Detection was made using anti-IgG antibodies conjugated with horseradish peroxidase (HRP) and 3.3′.5.5′-tetramethylbenzidine (TMB) chromogenic substrate. Prior to analysis, 50 μl of each samples were pooled and sequential dilutions were made to determine the appropriate dilution factor. For IgA measurements samples were diluted 1:5,000, and for IgG 1:3,00,000.

Absorbance was measured in duplicate samples on PowerWaveX-I microplate reader (Bio-Tek instruments, Winooski, VT, United States) at 450 nm. Standard dilutions were plotted and a smooth curve was drawn with a four-parameter logistic equation algorithm using the software provided by the microplate reader (KC4 v3.0 witk PowerReports^TM^, Bio-Tek instruments, Winooski VT, United States). Analyte concentrations in ng/mL were detected from the standard curve.

### Murine Study

#### Ethics Statement

This study was carried out in accordance with the EU directive 2010/63/EU on the protection of animals used for scientific purposes and the Danish Animal Experimentation Act (LBK 1306 from 2311/2007 with the 2010 amendments). The study was approved by the Animal Experimentation Inspectorate, Ministry of Food and Environment, Denmark.

#### Experimental Design

40 5-week-old barrier bred male BALB/cBomTac mice (Taconic, Lille Skensved, Denmark) were used for a 42-day study. The mice were allowed seven days of acclimatization (Day −7 to Day 0). Day of study start was considered as Day 0 and mice were terminated by cervical dislocation over two days at Day 41 and 42. All mice were co-housed in open cages at 21 ± 1°C with a 12-h light-time schedule with ad libitum access to food and bottled water. The mice were fed ad libitum Altromin 1324 maintenance diet (cereal based) (Altromin Gmbh, Lage, Germany) with slight modifications in the diet of the test-diet group as described below. The animals were randomly placed into four groups with ten mice in each: Group 1: inoculation-test group (mice inoculated with microbiota from test foals), Group 2: inoculation-control group (mice inoculated with microbiota from control foals), Group 3: test-diet group (mice receiving feed Altromin 1324 with test supplement added), and Group 4: control group (mice receiving Altromin 1324 without test supplement). During the acclimatization week, the mice in the inoculation-test group and inoculation-control group received Ampicillin (Ampivet, Boehringer Ingelheim, Denmark) 1 g/L through drinking water, to reduce their own microbiota prior to inoculation as previously shown to be efficient in microbiota transfer studies (Ellekilde et al., 2014b; Lundberg et al., 2016). Hereafter, they received a bacterial suspension prepared from fecal samples from either test or the control foals. The inoculation-test group received a suspension prepared from the foals fed the test supplement and mice in the inoculation-control group received a suspension prepared from the control foals. The suspensions were given by oral gavage on Day 0 and Day 7. Mice in the test supplement group received standard rodent diet Altromin 1324 (Brogaarden, Denmark) coated with the oligosaccharide supplement from Day 0 and until termination. The fourth group served as a baseline control and received Altromin 1324 diet without modifications.

The bacterial suspension given to the inoculation groups was prepared from pooled 30 g subsample taken from the Day 49 sample from three foals on test supplement and three control foals, respectively. The samples were randomly chosen between the foals in each group, however, foals from mares who had received antibiotics during parturition was excluded. Day 49 was chosen as the time of sampling from the foals as a significant difference in composition was present between the test and control foals at this time. Samples were pooled with 120 mL water in a stomacher bag (Stomacher 400 classic strainer sterile lab blender bag, Seward Ltd., West Sussex, United Kingdom) and processed in a stomacher (Seward 80 BA 7020 Stomacher Lab Blender, Seward Ltd., West Sussex, United Kingdom) for 2 min at maximum speed. The fecal slurry was extracted into 10 mL Falcon tubes and used for oral gavage.

#### Sample Collection

The mice were anesthetized by a mixture of Hypnorm^TM^ (VetPharma, United Kingdom) and midazolam (Roche A/S, Hvidovre, Denmark) (0.2 mL subcutaneously in a 1:1:2 water solution). Immediately prior to euthanasia blood was collected from the retro-bulbar sinus using a micro-hematocrit capillary tube (Thermo Fisher Scientific, Denmark). Hereafter, the mesenteric lymph nodes (MLN) were sampled and placed in RNAlater (Sigma, Life Science, St. Louis, MO, United States). Ileum, cecum, and colon were emptied and intestinal contents were snap frozen using dry ice and then stored at −80°C until bacterial DNA extraction, 1 cm samples of intestinal tissue from ileum, cecum and colon were placed in RNAlater and stored at −80°C for later gene profiling. Total DNA was extracted from the intestinal content as described for the equine fecal samples but without the stomacher and centrifuge pre-step. 16S rRNA gene amplicon sequencing was performed as lined out above.

#### RNA Isolation and cDNA Synthesis

RNA was extracted from MLNs and intestinal tissue from the ileum and cecum. The tissues were thawed on ice and transferred to FastPrep^®^ tubes (MP Biomedical, Illkirch, France) containing 0.6 mg acid-washed glass beads (<106 μm. Sigma Life Science, St. Louis, MO, United States), 500 μl MagMax^TM^ Lysis/binding solution concentrate (Thermo Fisher Scientific, Denmark) and β-Mercaptoethanol (Sigma Life science, St. Louis, MO, United States). The tissues were completely homogenized by three rounds with a FastPrep^®^-24 (MP Biomedical, United States) at speed 6.5 m/s for 45 s and hereafter stored at −20°C for 48 h. For RNA extraction MagMax^TM^-96 Total RNA Isolation Kit (Cat. No. AM1830, MA, United States) and the MagMax^TM^ Express magnetic particle processor (Thermo Fisher Scientific, Herlev, Denmark) was used with 100 μl homogenate input. RNA concentration and quality was measured using 2000 NanoDrop Spectrophotometer (Thermo Fisher Scientific, Waltham, MA, United States) before preparing dilutions of the RNA samples to be used for the following complementary DNA (cDNA) synthesis. The cDNA synthesis was carried out using High Capacity cDNA Reverse Transcriptase Kit (Cat. No. 4368814, Applied Biosystems, Foster City, CA, United States) according to suppliers protocol, with approximately 500 ng RNA input, and 2720 Thermal Cycler (Applied Biosystems, CA, United States).

#### Gene Expression by qPCR

Gene expression of *18S, Il10, Il12b, Tnfa*, and *Foxp3* was analyzed in the MLN, ileum, and caecum using TaqMan probes (Assay ID: 18S – 4333760F, Foxp3 – Mm00475162_m1, TNF-α – Mm00443258_m1, IL-10 – Mm0043961616_m1, IL-12b – Mm01288989_m1; Applied Biosystems, CA, United States) and TaqMan Fast Universal PCR Master Mix (2X), cDNA was amplified in singles and Non Template Controls were included for each investigated gene. The amplification data was analyzed using the StepOne v 2.3 software (Applied Biosystems, Thermo Fisher Scientific, Denmark) to obtain threshold cycle (C_*T*_) values. Quality check of the amplification data was performed and samples flagged NOSIGNAL (no detectable level of fluorescence), NOISE (the background fluorescence in the well exceeds the limit), and BLFAIL (baseline cannot be fitted for the well) were excluded from the analysis.

### Statistical Analysis

Quantitative Insight Into Microbial Ecology open source software packages (1.7.0, 1.8.0, and 1.9.1) (Caporaso et al., 2010; McDonald et al., 2012) were used for the analysis of the sequencing data. The script compare_alpha_diversity.py was used to generate richness indices: observed species and Shannon index. For the equine study, a two-sample *t*-test using non-parametric Monte-Carlo permutations to calculate the p-value was used to assess the differences between the test and control group at the different time-points. For the murine study, normality of alpha diversity was assessed by a Shapiro-Wilks normality test and data not following a Gaussian distribution were analyzed by a Kruskall–Wallis test using GraphPad Prism7 (GraphPad Software Inc., La Jolla, CA, United States). In both studies, UniFrac distance metrics were calculated from subsampled OTU tables (10,000 reads/sample) and visualized with principal coordinates analysis (PCoA) plots. The differences in ordination between categories in the murine study were tested using the analysis of similarity test (ANOSIM). In the equine study, the differences in taxa abundance between time points were estimated with a statistic framework: analysis of composition of microbes (ANCOM) based on non-normalized OTU-table summarized to the species level (Mandal et al., 2015). Day 7 samples of IgA and IgG values in the test and control group were compared with a nonparametric Mann–Whitney *U* test and when no differences were found between the two groups, samples from Day 28 and 49 were normalized to Day 7 levels and compared by repeated measures ANOVA test. Weekly pH was likewise compared by repeated measures ANOVA. Fold changes in gene expression were calculated according to the ddCt method. The expression of the target genes, calculated as the C_*T*_, were normalized to the reference gene 18S [DC_*T(sample)*_ = C_*T(target)*_ – C_*T(reference)].*_ Fold changes were calibrated to the control group an expressed as 2^–*ddCT*^ where ddC_*T*_ = DC_*T(sample)*_ – DC_*T(control group)*_. Graphs for gene expression were plotted using the fold changes, while calculations were based on ddC_*T*_ values. Normality of cytokine data was checked by a Shapiro-Wilks normality test and data was analyzed by one-way ANOVA with post-hoc Tukey’s test. Data that did not follow a Gaussian distribution were analyzed by a nonparametric Mann–Whitney *U* test. A Spearman Rank correlation test was performed using SAS University Edition (SAS Institute Inc., NC, United States). Bacterial abundances in the cecum of mice were correlated to relative gene-expression in the cecal mucosa and MLN. Bacterial abundances in the colon were correlated to relative gene-expression in the MLN. Only species previously shown to correlate with regulatory immunity markers in horses were included in the analysis (Lindenberg et al., 2019a). *P*-values were FDR corrected. In all tests *p*-values below 0.05 were regarded significant.

## Results

### Mannanoligosaccharides and FOS Feeding Induced Significant Differences in the Foal Gut Microbiota Driven by Changes in Abundances of *Akkermansia* and *Proteobacteria*

**FIGURE 1:**
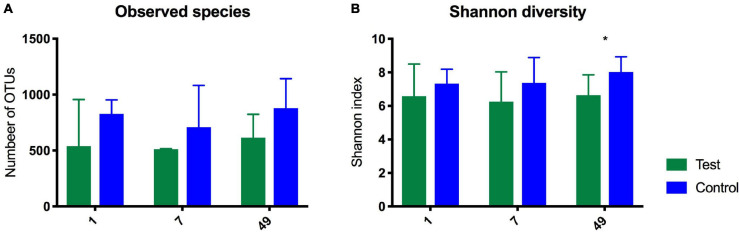
Diagrams showing observed species **(A)** and Shannon Index **(B)** as determined by 16S rRNA gene amplicon sequencing in the gut of foals fed either a test supplement enriched with oligosaccharides or a control diet on Day 1, 7, and 49 after birth. Shown as mean +SD of number of observed OTUs (qualitatively) and on the Shannon index (quantitatively). 49 days after birth there was a significant difference on the Shannon index. *P = 0.04.

**FIGURE 2:**
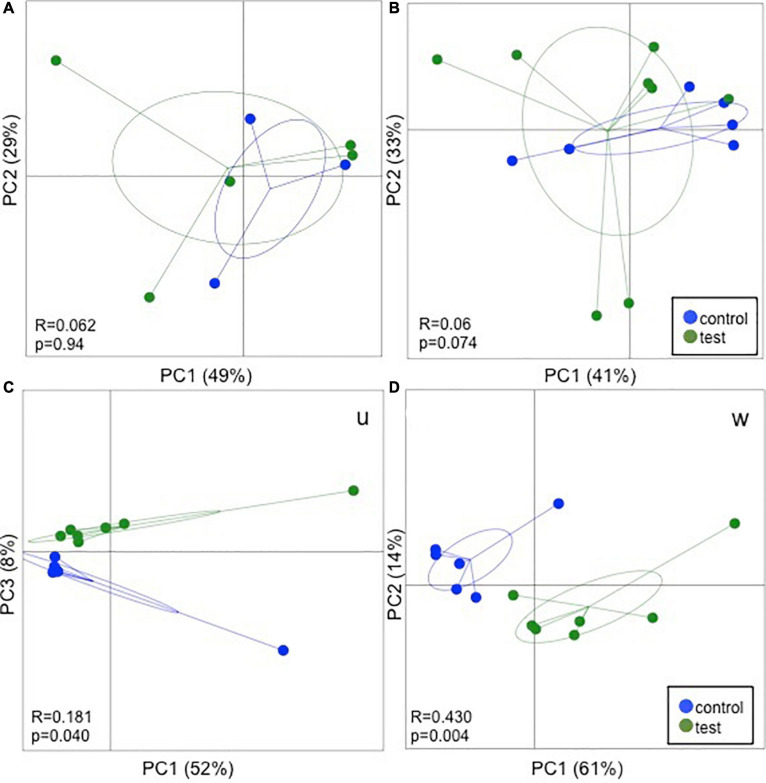
16S rRNA gene amplicon sequencing results fecal samples from foals fed either a test supplement enriched with oligosaccharides or a control diet at Day 1 **(A)**, Day 7 **(B)**, and Day 49 **(C,D)** post parturition. Principal coordinates analysis (PCoA) plots based on weighted UniFrac distance matrices show no clear separation due to the treatment on Day 1 and 7. On Day 49 significant differences were observed in both unweighted (U) and weighted (W) UniFrac distance. ANOSIM results are presented in the bottom left corner of each plot.

**FIGURE 3:**
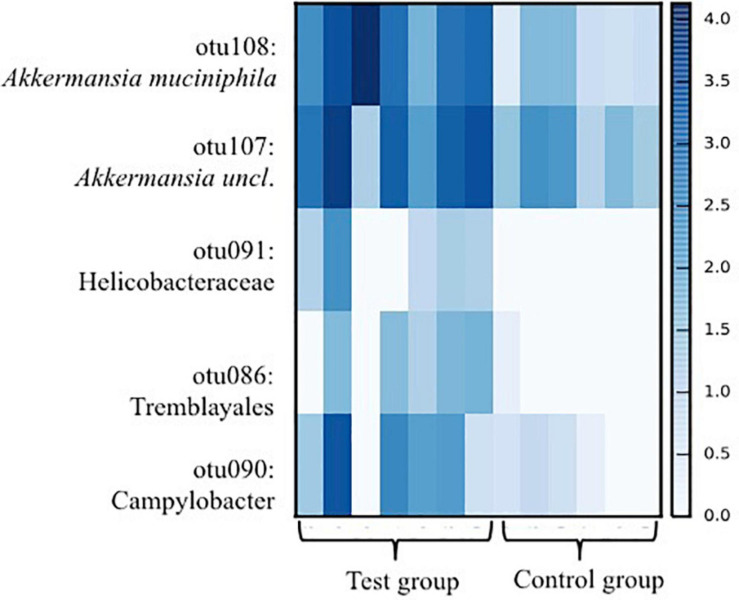
Heat-map showing taxa in which significant differences (*p* < 0.05) between foals fed either a test supplement enriched with oligosaccharides or a control diet as detected in ANCOM analysis based on 16S rRNA gene amplicon sequences from fecal samples collected from foals 49 days after birth. Scale on the right indicates log abundance.

The species richness increased in both the test and control foals from birth until Day 49 (Figures 1A,B), Alpha diversity tended to be lower in the test group compared with the control group and was significantly lower 49 days after birth as determined by the Shannon index, which takes the richness and evenness of each of the observed species into account (Figure 1B). PCoA plots based on unweighted, weighted and generalized UniFrac distances showed no clear separation between test and control foals on Day 1 or Day 7, indicating a very heterogeneous composition in the first week of life. Examples of weighted UniFrac distances based PCoA plots are presented in Figures 2A,B. Significant differences were detected 49 days post-partum based on both unweighted (Figure 2C) and weighted UniFrac distances (Figure 2D). Community analysis of the differences at Day 49 revealed significant differences between the test and control group, and that especially members of the two phylae Proteobacteria and Verrucomicrobia were responsible for driving the differences (Figure 3). Among the Proteobacteria three different taxa belonging to order *Tremblayales*, family Helicobacteraceae and genus *Campylobacter* were significantly more abundant in the test group compared to the control group (Figure 3 and Supplementary Figure 1). From the phylum Verrucomicrobia, *Akkermansia* ssp. were significantly more abundant in the test group compared to the control (Figure 3 and Supplementary Figure 1). No significant differences were found in abundance of any of the other 14 taxa, which we have previously correlated to regulatory immunity (Lindenberg et al., 2019a). There were no detectable changes in the unweighted, weighted, or generalized UniFrac distances of the mare gut microbiota at either Day −7, 1, 7, or 49 during the feeding period (data not shown), nor were there any significant differences either qualitatively or quantitatively in alpha diversity between the test and control mares.

### Inoculation of Mice With Microbiota From Test Foals Increased Markers of Regulatory Immunity in the Mucosal Membrane, Microbial Diversity, and Abundance of Species Shown to Induce Regulatory Immunity in Horses

**FIGURE 4:**
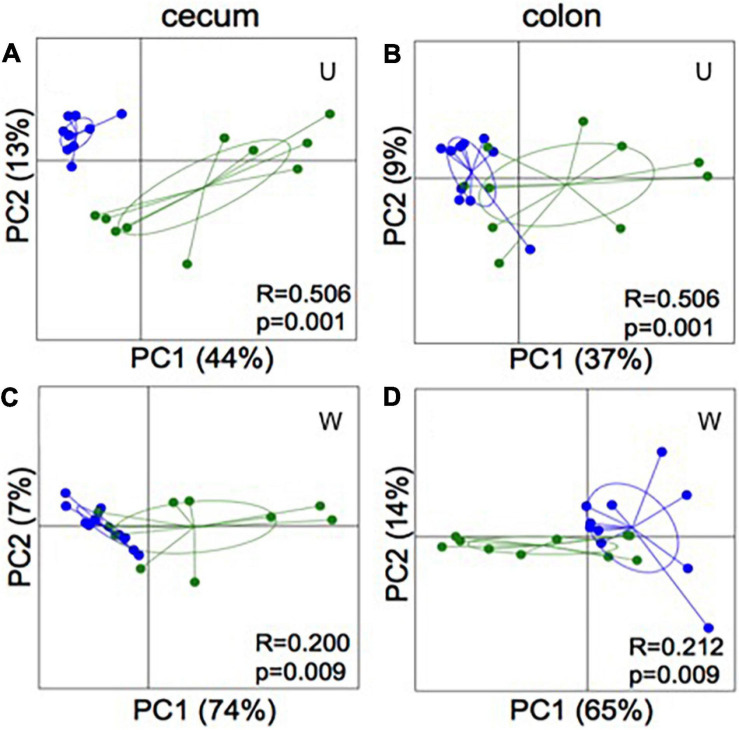
16S rRNA gene amplicon sequencing of cecum **(A,C)** and colon **(B,D)** content of mice inoculated with microbiota from foals fed either a test supplement enriched with oligosaccharides (green) or a control diet (blue). PCoA plots based on weighted (W) and unweighted (U) UniFrac distances show significant separation (ANOSIM: bottom right coroner) between groups in both compartments of the gut.

**FIGURE 5:**
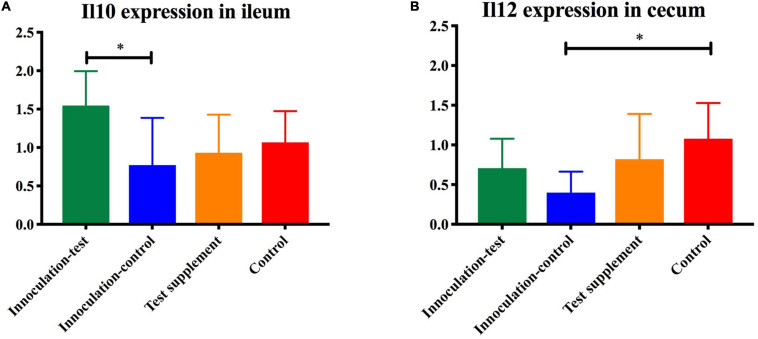
Gene-expression of *Il10* **(A)** and *Il12* **(B)** measured by qPCR in the ileum of mice inoculated with microbiota from foals fed either a test supplement enriched with oligosaccharides (green) or a control diet (blue) or fed a mouse diet with (orange) or without (red) oligosaccharides. The expression of *Il10* was significantly increased (*p* < 0.05) in the inoculation-test group compared with the inoculation-control group and the expression of *Il12* was significantly decreased in the control-inoculation group compared to the control, indicated by ^∗^.

**FIGURE 6:**
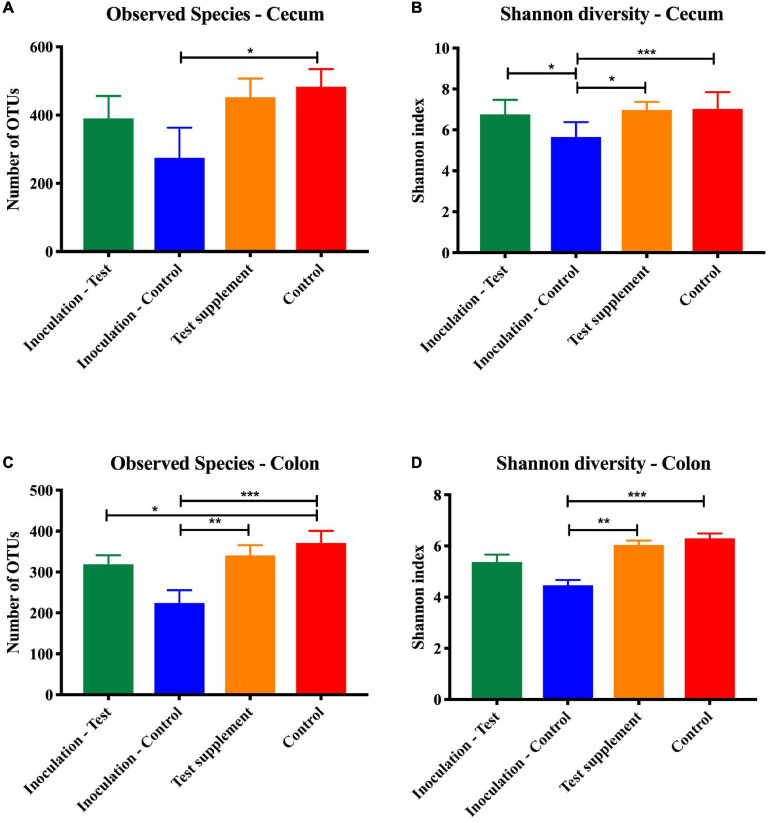
Alpha diversity measured on 16S rRNA extracted from cecal and colon content at termination of mice inoculated with microbiota from foals fed either a test supplement enriched with oligosaccharides (green) or a control diet (blue) or fed a mouse diet with (orange) or without (red) oligosaccharides. Shown qualitatively as number of observed OTUs (mean, SD) and quantitatively by the Shannon index (mean, SD). When compared by one-way ANOVA significant differences were found in the colon content but not in the cecum marked as *p < 0.05, **p < 0.01, and ***p < 0.005.

Analysis of similarity revealed significant differences in the microbial composition between the inoculation test and inoculation control group in both cecum (unweighted *p* = 0.001 and weighted *p* = 0.009) (Figures 4A,C) and colon (unweighted *p* = 0.001 and weighted *p* = 0.009) (Figures 4B,D). Differences were observed between the inoculation test and inoculation control groups within the orders Clostridiales, Bacteroidales, Erysipelotrichaceae, Lactobacilliales, and Streptophyta, but none were significant after FDR correction (Table 1). In the ileac mucosa expression of the *Il10* gene was significantly increased in the inoculation test group compared to the inoculation control group (Figure 5A). In the cecal mucosa expression of *Il12* was significantly lower in the inoculation control group compared to the control (Figure 5B). Generally, the alpha-diversity was lower in both the inoculation test and inoculation control groups when compared to the test-diet and control groups (Figure 6). The inoculation control group had a significantly lower diversity (Shannon index) in the cecum than the inoculation test group (Figure 6B). The inoculation test group had significantly lower abundance of observed species in the colon than the control group (Figure 6C). Several differences were found in the relative abundance between the two inoculation groups, of special interest were increased abundances in the test inoculation group of five taxa that we have previously correlated to anti-inflammatory signals in horses (Lindenberg et al., 2019a). These were unclassified members of Clostridiales and Ruminococcaceae, *Ruminococcus, Oscilospira* and *Coprococcus*. In the mice we did not observe any of the correlations between relative bacterial abundance and gene-expression previously found in the horses. Interestingly, several of the species, which we positively correlated to markers of regulatory immunity in horses, had a negative correlation to the markers of regulatory immunity measured in mice (Table 2), indicating host specific interactions.

**TABLE 1:** Differences analyzed with ANOVA in relative abundance of OTUs between mice inoculated with microbiota from foals fed either a test supplement enriched with oligosaccharides or a control diet or fed a mouse diet with or without oligosaccharides.

Taxa								
Phylum	Class	Order	Family	Genus	Inncoulation-test mean	Innoculation-control mean	*P*-value	FDR corrected
Bacteroidetes	Bacteroidia	Bacteroidales	Bacteroidaceae	Bacteroides	0.0047	0.3835	0.0055	0.1761
Firmicutes	Clostridia	Clostridiales	Ruminococcaceae; Other		0.0090	0.0012	0.0060	0.0953
Firmicutes	Clostridia	Clostridiales	Clostridiaceae	*Candidatus Arthromitus*	0.0025	0.0005	0.0064	0.0685
Firmicutes	Erysipelotrichi	Erysipelotrichales	Erysipelotrichaceae	Coprobacillus	0.0000	0.0003	0.0073	0.0584
Bacteroidetes	Bacteroidia	Bacteroidales	[Odoribacteraceae]	Odoribacter	0.0088	0.0000	0.0107	0.0684
Firmicutes	Bacilli	Lactobacillales	Lactobacillaceae	Lactobacillus	0.1712	0.0562	0.0374	0.1997
Cyanobacteria	Chloroplast	Streptophyta			0.0070	0.0022	0.0381	0.1740
Firmicutes	Erysipelotrichi	Erysipelotrichales	Erysipelotrichaceae		0.0003	0.0008	0.0512	0.2047
Firmicutes	Clostridia	Clostridiales			0.2259	0.1334	0.0555	0.1974
Bacteroidetes	Bacteroidia	Bacteroidales	[Barnesiellaceae]		0.0000	0.0104	0.0721	0.2308
Firmicutes	Erysipelotrichi	Erysipelotrichales	Erysipelotrichaceae	[Eubacterium]	0.0001	0.0008	0.0811	0.2358
Firmicutes	Clostridia	Clostridiales	Ruminococcaceae	Oscillospira	0.0145	0.0071	0.1130	0.3014
Firmicutes	Clostridia	Clostridiales	Lachnospiraceae	Dorea	0.0009	0.0020	0.1169	0.2877
Proteobacteria	Gammaproteobacteria	Enterobacteriales	Enterobacteriaceae		0.0002	0.0018	0.1424	0.3255
Firmicutes	Clostridia	Clostridiales	Lachnospiraceae;Other		0.0183	0.0118	0.1509	0.3218
Tenericutes	Mollicutes	RF39			0.0006	0.0149	0.1668	0.3336
Firmicutes	Clostridia	Clostridiales	Ruminococcaceae	Ruminococcus	0.0035	0.0018	0.1675	0.3153
Firmicutes	Clostridia	Clostridiales	Lachnospiraceae	Balutia	0.0002	0.0006	0.1802	0.3203
Bacteroidetes	Bacteroidia	Bacteroidales	Rikenellaceae;Other		0.0273	0.0143	0.1873	0.3155
Bacteroidetes	Bacteroidia	Bacteroidales	Rikenellaceae		0.3111	0.1923	0.1912	0.3059
Bacteroidetes	Bacteroidia	Bacteroidales	Porphyromonadaceae	Parabacteroides	0.0001	0.0002	0.2231	0.3400
Firmicutes	Clostridia	Clostridiales	Ruminococcaceae; other		0.0009	0.0018	0.2372	0.3451
Firmicutes	Clostridia	Clostridiales	Lachnospiraceae		0.0787	0.0577	0.2551	0.3549
Firmicutes	Bacilli	Lactobacillales	Streptococcaceae	Streptococcus	0.0003	0.0001	0.2555	0.3407
Firmicutes	Clostridia	Clostridiales	Lachnospiraceae	[Ruminococcus]	0.0224	0.0157	0.2722	0.3484
Firmicutes	Clostridia	Clostridiales	Ruminococcaceae		0.0115	0.0080	0.4759	0.5858
Firmicutes	Clostridia	Clostridiales	Lachnospiraceae	Oribacterium	0.0000	0.0001	0.5555	0.6584
Firmicutes	Clostridia	Clostridiales	[Mogibacteriaceae]		0.0001	0.0001	0.5615	0.6417
Bacteroidetes	Bacteroidia	Bacteroidales	Prevotellacea	Prevotella	0.0001	0.0001	0.6681	0.7372
Firmicutes	Clostridia	Clostridiales	Lachnospiraceae	Coprococcus	0.0015	0.0014	0.7616	0.8124
Unassigned					0.0003	0.0003	0.7904	0.8159
Bacteroidetes	Bacteroidia	Bacteroidales	S24–7		0.0768	0.0772	0.9901	0.9901

*The probability (*p*-value) was calculated based on 1,000 subsampled OTU tables rarefied to 10.000 per sample. The false discovery rate (FDR) represents the probability after correction with FDR where the raw p-values are firstly ranked from low to high and then each p-value is multiplied by the number of tests divided by this rank. “Unclassified” stands for taxa having no official taxonomy in the database. Taxa denoted as “Other” indicates ambiguity in the assignment, meaning that more than one taxon could be assigned to this cluster at given taxonomic level. Taxa mentioned in the square brackets indicate a proposed taxonomy.*

**TABLE 2:** Spearman rank correlation analysis between bacterial abundances in the cecum and colon of species previously shown to induce regulatory immunity in horses and gene-expression measured in the cecal mucosa and mesenteric lymph nodes of mice inoculated with fecal material from foals fed a control diet and from mice fed either a test supplement enriched with oligosaccharides or a control diet.

Group	Compartment	Taxa	Gene and compartment of gene expression	Correlation coefficient	*P*-value	FDR corrected
Inoculation control	Cecum	Clostridiales	tnfa cecum	−0.8	0.0096	0.0384
		Clostridiales	foxp3 cecum	−0.70909	0.0217	0.0421
		Lachnospiraceae	tnfa cecum	−0.81667	0.0072	0.0384
		Lachnospiraceae	foxp3 cecum	−0.70909	0.0217	0.0421
		Ruminococcaceae	foxp3 cecum	−0.73557	0.0153	0.0421
		Oscillospira	foxp3 cecum	−0.816510	0.0012	0.0096
		Ruminococcus	foxp3 cecum	−0.6931	0.0263	0.0421
Test supplement	Cecum	Lachnospiraceae	tnfa mln	−0.82468	0.0062	0.0386
		Coprococcus	tnfa mln	−0.081452	0.0075	0.0384
		Ruminococcus	il10 mln	−0.86667	0.0012	0.0096
		Ruminococcus	foxp3 mln	−0.66061	0.0376	0.3008
	Colon	Ruminococcus	il12b mln	−0.86697	0.0012	0.0096
Control	Cecum	Clostridiales	tnfa cecum	−0.86325	0.0027	0.0384
		Lachnospiraceae	tnfa cecum	−0.82468	0.0062	0.0386
		Coprococcus	tnfa cecum	−0.81452	0.0075	0.0424

### The Test Supplement Did Not Affect Fecal pH in the Mares or IgA and IgG in the Foals

**FIGURE 7:**
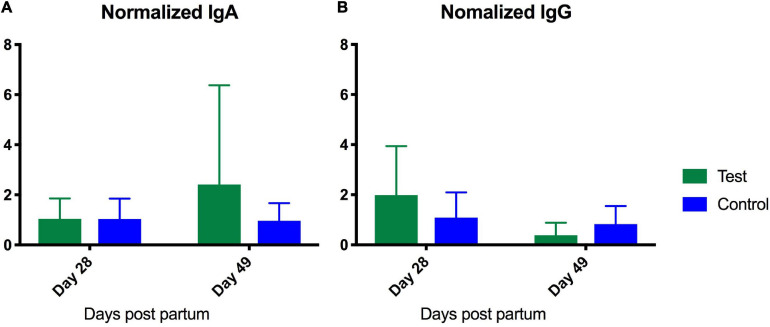
Enzyme-linked immunosorbent assay analysis of serum content of IgA **(A)** and IgG **(B)** from foals fed either a test supplement enriched with oligosaccharides or a control diet. *X*-axis shows levels (ng/mL) measured at Day 28 and 49 normalized to levels measured on Day 7 as baseline. A repeated measures test investigating the development from day 7 to 40 revealed no significant differences.

The test supplement was accepted and ingested well, and none of the mares developed clinical signs indicative of side effects, i.e., colic, diarrhea, inappetence, or depression. No significant differences in fecal pH were detected. The mean at the different time points varied between 6.16 and 6.68 for the test group and 6.27–6.59 for the control group. No significant differences in immunoglobulin levels were detected between the groups at Day 7. Samples were normalized to Day 7 levels, which served as base-line, to monitor the development from Day 7 and onward. No significant differences were detected in the repeated measurements test. The IgA levels tended to increase in the test group but remained on the same level in the control group (Figure 7A). IgG levels decreased in both groups (Figure 7B).

## Discussion

As hypothesized a mixture of MOS and FOS promoted the growth of anti-inflammatory bacteria in the equine GIT. As significant increases were observed in the abundance of *Akkermansia* spp. The anti-inflammatory bacteria were not, as hypothesized passed on to colonize the foals, as no differences in microbial composition were detected in the foals before Day 49, at which time they were eating the mares’ food in addition to drinking milk. This indicates that ingestion of oligosaccharides from the diet was the primary and not as hypothesized an additive inducer of differences between test and control foals. It was noted that all foals ingested the mares feed but the amounts ingested was not measured thus it is not possible to say exactly how much of the supplement each foal had. In the early life foals are also coprophagic, thus microbiota may also have been passed on from mare to foal this way. However, as we did not detect any changes in the mares’ gut microbiota composition, we do not attribute this mode of microbiota transfer as the sole source of the differences in gut microbiota detected in the two groups of foals. In addition, no differences were observed between the gut microbiota composition of the test and control mares, suggesting that vertical transfer of bacteria from mares to foals have not contributed to the increase in *Akkermansia* spp. As hypothesized immune responses were influenced in the foals.

One *Akkermansia* spp., *A. muciniphila* which we have previously shown to be abundant in the equine gut (Lindenberg et al., 2019a) is known to inhabit the mucin layer and to help uphold the gut balance by conversion of mucin to the beneficial SCFAs acetate and propionate (Derrien et al., 2004, 2008). We have previously shown a positive correlation between Verrucomicrobia to which *Akkermansia* belongs and expression of the regulatory T-cell transcription factor *FOXP3* (Lindenberg et al., 2019a). In that study, *Akkermansia* was far the most abundant member of the phylum suggesting that it was responsible for the majority of the immunological interaction, which may suggest that the unclassified *Akkermansia* may also have anti-inflammatory effects. Reduced abundances of *A. muciniphila* have been associated with inflammatory diseases such as inflammatory bowel disease (IBD) in humans (Png et al., 2010), colitis, T2D, and obesity in mice (Kang et al., 2013; Ellekilde et al., 2014a). Increasing the abundance of *A. muciniphila* significantly improved glucose metabolism, decreased pro-inflammatory cytokines IL-1b and IL-16 and increased levels of Tregs in diet induced obese mice (Shin et al., 2014). Vancomycin treatment of mice early in life, lead to higher abundances of *A. muciniphila* and significantly lower type 1 diabetes incidence (Hansen et al., 2012a) suggesting that increases in *A. muciniphila* in the early life may reduce the incidence of certain diseases later on. Of particular interest for this study are the findings by Everard et al. (2013), who found a direct link between FOS feeding, increased the abundance of *A. muciniphila*, and significant improvements in glucose- and insulin metabolism, mucosal barrier function, adipose tissue inflammation, reduced fat gain, and endotoxemia in mice. Their results support our results and hypothesis that oligosaccharides induce anti-inflammatory bacteria, which could positively impact the immune system and disease development.

In addition to *Akkermansia* spp. significant increases were observed within *Campylobacter*, and *Helicobacter* spp. in the test group compared to the control group. Certain species of *Campylobacter* are known pathogens and the cause of gastrointestinal disease (Van Vliet and Ketley, 2001). We and others have previously found this taxa in healthy horses suggesting that this might be a non-pathogenic species or that low abundances are part of the commensal equine microbiota (Moriarty et al., 2015; Lindenberg et al., 2019a), however, this should be investigated further, before non-pathogenicity can be assumed. We and others have also previously found *Helicobacter* spp. in the gut of healthy foals (Moyaert et al., 2009; Lindenberg et al., 2019b). Its presence has not been associated with any macro- or micro-pathologies in horses (Moyaert et al., 2007) suggesting it is also a part of the normal transient microbiota that initially colonizes the equine gut, however, pathogenic *Helicobacter* species exists, and further studies should determine the species of the *Helicobacter* in the equine GIT. Further, the betaproteobacteria *Tremblayales*, which so far has not been associated with either pathological or beneficial properties in healthy foals was detected (Lindenberg et al., 2019b). Proteobacteria contain LPS in the membrane, which in adults has been shown to induce endotoxemia and inflammation (Carvalho et al., 2012), but studies have shown that a lipopolysaccharide (LPS) boost early in life may lead to improved regulatory immunity (Lotz et al., 2006; Bendtsen et al., 2017). It may be speculated that by consuming mucin. *Akkermansia* may have reduced the mucosal layer. Such a mucosal layer reduction may facilitate interaction between bacterial LPS and the mucosal immune system thereby leading to an improved stimulation and subsequently improved the potential for inducing regulatory immunity (Bendtsen et al., 2018). Low abundances of both *Campylobacter* and *Tremblayales* have also been found in the colonic mucosa of healthy weanling piglets (Grosu et al., 2019), indicating that their presence may be normal in the microbiota of young animals. All foals who received the test supplement were healthy and did not show any gastro-intestinal symptoms. However, further studies determining the subspecies of both *Campylobacter, Helicobacter* and *Tremblayales* would be beneficial in determining whether increased abundance might have undesired effects on the gastro-intestinal system.

Mannanoligosaccharides fed to mares before parturition has previously been found to increase the levels of IgG and IgA in colostrum (Spearman and Ott, 2004). It may be speculated whether the small differences in IgA and IgG levels detected between the test and control foals indicate that the oligosaccharides may affect immunoglobulin levels, but further studies would be needed to warrant this. Future studies should investigate whether this effect is mediated through oligosaccharides increasing the colostrum immunoglobulin levels, which would then give reason to feed the mares pre-partum. Oligosaccharides may through their ability to stimulate the growth of specific species work as a “stabilizing agent” in the same way as it stabilizes the microbiota during diet changes (Respondek et al., 2008) and hence lead to the reduced alpha diversity observed in the test foals in this study. A similar effect was observed in a study comparing the gut microbiota of human infants fed an iso-caloric formula with or without galactooligosaccharides (GOS) (Matsuki et al., 2016). Oligosaccharides are routinely used in equine research to induce hindgut acidosis and subsequently laminitis, but we observed no detectable difference in pH between the test and control mares indicating that the test supplement in the given dose does not induce hindgut acidosis and as such is safe to use.

As it was not possible to obtain intestinal biopsies from the foals, we aimed to assess the local immunological effects in inoculated mice, which is also done in studies of human diseases (Zachariassen et al., 2017). Significant differences in the microbial composition were observed between the mice inoculated with microbiota from the test and control foals respectively, indicating that it is possible to transfer gut microbial differences from horses to mice. The microbial composition was not identical between test foals and inoculation test mice and control foals and inoculation control mice respectively, which is most likely due to the general loss of diversity during inoculation procedures (Ellekilde et al., 2014b), a reduced colonization rate (Lundberg et al., 2017) and differences observed in xeno-inoculations (Chung et al., 2012). The increased level of *Akkermansia* spp. was not transferred to the mice, but six genera, which we have previously correlated to regulatory immunity in horses, were found in increased abundances in the inoculation test group compared to the inoculation control group (Lindenberg et al., 2019a). This indicates that the microbial composition from the test foals had a greater potential of inducing regulatory immunity. The significantly increased expression of *Il10* encoding the regulatory IL-10 cytokine in the ileum of the inoculation test group supports this hypothesis. Inoculation procedures and pre-treatment with antibiotics are associated with loss of species richness (Ellekilde et al., 2014b), and this is most likely the cause of the generally lower alpha-diversity in both the inoculation test and inoculation control mice. The lower alpha diversity may also explain the significantly lower *Il12* expression in the inoculation control group compared to the test supplement control group, *Il12* encode the pro-inflammatory interleukin 12 (IL-12) secreted by dendritic cells to stimulate the formation of T-helper 1 (Th1) cells that are stimulated by microbiota, and a reduced species richness in the inoculation control group may also have reduced the overall immune stimulation. The differences between the correlation analysis in the mice and the previous results from horses may be explained by the age of the mice at the time of inoculation. The mice were inoculated at 6 weeks of age at which time the window of opportunity for development of oral tolerance has closed (Hansen et al., 2012b). In the previous study, the horses were assumed to have acquired the same species as foals, and thereby have developed oral tolerance toward them. Optimally the inoculation should, therefore, have been performed on animals under 3 weeks of age, which can be done by inoculation of the mothers to have the pups born with the microbiota (Zachariassen et al., 2017). This should eventually be pursued in future horse to mouse inoculations. Additionally, to the potential regulatory immunity inducing taxa we also observed increased abundances of *Candidatus Arthromitus* also known as segmented filamentous bacteria (SFB) in the inoculation-test mice. We did not observe any SFBs in the foals, so the increase is not likely to be a direct effect of inoculation with SFB. However, the altered microbial composition may have changed the living conditions for certain resident bacteria and caused a bloom in SFBs. After ampicillin treatment a few species of resident bacteria may propagate in mice (Castro-Mejia et al., 2018). SFBs selectively stimulate the differentiation of T-helper 17 (Th17) cells, which are required for a normal immune balance, and SFB status has been shown to reduce the incidence of type 1 diabetes (Kriegel et al., 2011) and increase the severity of IBD (Ivanov et al., 2008, 2009) in mice. A further investigation into this increased abundance should, therefore, be conducted, preferably by also assessing the level of IL-17 between the two groups.

It should be noted that the applied oligosaccharides were extracted from yeast cell walls, and these also contain the polysaccharides known as β-Glucans (Fesel and Zuccaro, 2016), and therefore traces of these have been present in our preparation. β-Glucans from fungi exhibit a broad spectrum of biological activities including anti-tumor, immune-modulating and anti-inflammatory properties (Du et al., 2015), which may also have added to the effects observed in this study.

In conclusion, our oligosaccharide containing test supplement increased the abundances of *Akkermansia* spp. the presence of which is known to be associated with improved mucosal health, lower inflammatory level, and higher levels of regulatory immunity, which may reduce incidence or severity of inflammatory diseases later on. The test group also had increased abundance of *Campylobacter, Helicobacter*, and *Tremblayales*, which we believe to be part of the transient microbiota of young foals, but further studier should be carried out to determine this, as spp. are also known to be pathogenic. The effects of the test supplement seemed to be caused by intake of the diet by the foals and direct microbial stimulation by the oligosaccharides. Mouse inoculations showed an anti-inflammatory potential of the gut microbiota from the oligosaccharide fed test foals.

## Data Availability Statement

The datasets presented in this study can be found in online repositories. The names of the repository/repositories and accession number(s) can be found below: https://www.ncbi.nlm.nih.gov/bioproject/, ID PRJNA700065, https://figshare.com/articles/dataset/14604804, and https://figshare.com/articles/dataset/13713460.

## Ethics Statement

The animal study was reviewed and approved by the Danish Animal Experiments Expectorate (Murine study). Written informed consent for participation was not obtained from the owners because the equine study was carried out as part of a long standing collaboration agreement between the stud farm and Brogaarden. The owners and staff were carefully informed and instructed prior to the study. Furthermore, all samples were collected as part of the stud farm’s routine health surveillance, by a certified veterinarian, which according to the Animal Experimentation Act (LBK No. 474 15/05/2014), should not be regarded as animal experimentations. All procedures were carried out according to The Danish Veterinary Act (Dyrlægeloven) no 48 of 11/01/2017.

## Author Contributions

FL, JF, HF, GG, DN, and AKH planned the experiment. FL collected the samples for the Equine study, performed the DNA extraction, and performed the ELISA analysis of the equine study. FL and DL collected the samples for the murine study. FL, LK, and AKH performed the sequencing. FL, LK, and DN performed the data analysis of the sequencing data. FL and DL performed the data analysis. DL performed the RNA extraction and qPCR of the murine study. FL, LK, and AH performed the statistics. All authors took part in drafting and approving the manuscript.

## Conflict of Interest

FL works at Brogaarden Diets. AKH declares that he has several industrial collaborations as further described on https://ivh.ku.dk/english/employees/?pure=en/persons/107126. The study received funding from Innovation Fund Denmark, who was not involved in the study design, collection, analysis, interpretation of data, the writing of this article or the decision to submit it for publication. The study received funding from Brogaarden which is the employer of FL, and was therefore involved in the study design and execution.

## Abbreviations

ANCOM: analysis of composition of microbes
ANOSIM: analysis of similarity
ANOVA: analysis of variance
cDNA: complimentary deoxyribonucleic acid
C_*T*_: threshold cycle
dC_*T*_: delta threshold cycle
ddC_*T*_: delta delta threshold cycle
DNA: deoxyribonucleic acid
ELISA: enzyme-linked immunosorbent assay
EMS: equine metabolic syndrome
FOS: fructooligosaccharides
Foxp3: forkhead box P3
GOS: galactooligosaccharides
GIT: gastro-intestinal tract
HMO: human milk oligosaccharides
HRP: horseradish peroxidase
IBD: inflammatory bowel disease
Ig: immunoglobulin
IL-10: interleukin 10
IL-12: interleukin 12
LPS: lipopolysaccharides
MOS: mannanoligosaccharides
OD: optical density
OTU: operational taxonomic unit
PCoA: principal component analysis
PCR: polymerase chain reaction
RNA: ribonucleic acid
rRNA: ribosomal ribonucleic acid
qPCR: quantitative polymerase chain reaction
SCFA: short chain fatty acids
SFB: segmented filamentous bacteria
TGF- β: transforming growth factor-beta
Th1: T-helper 1 cell
Th17: T-helper 17 cell
TMB: 3.3 ′ .5.5 ′ -tetramethylbenzidine
TNF- α: tumor necrosis factor alpha
Treg: regulatory T-cell
T2D: type 2 diabetes
18s: 18s ribosomal ribonucleic acid.
