# Supplementary material for: An Oligosaccharide Rich Diet Increases Akkermansia spp. Bacteria in the Equine Microbiota
Source: Front Microbiol. 2021 May 21;12:666039. doi: 10.3389/fmicb.2021.666039 (PMC8176217; doi:10.3389/fmicb.2021.666039)
Supplement: Supplementary file 1 [file Data_Sheet_1.PDF]

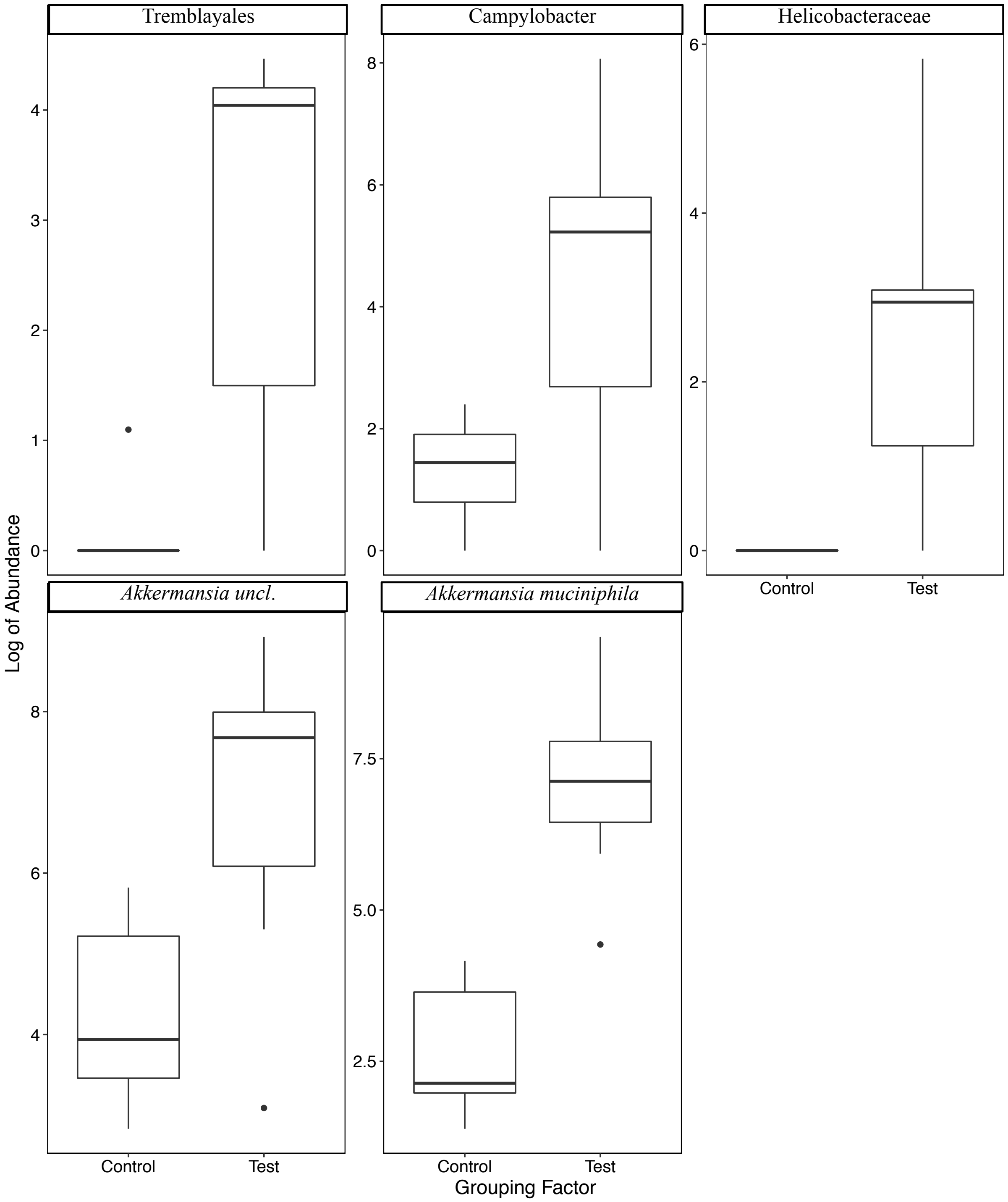

Supplementary Figure 1. Boxplot showing the log abundance median +/- quartiles (whiskers represent the 1.5 IQR and dots the extremes) of OTUs based on 16S rRNA sequences from fecal samples collected from foals fed either a test diet enriched with oligosaccharides or a control diet 49 days after birth. Only box plots of species that differed significantly (ANCOM, FDR < 0.05) between the test and control foals.

Optimal nr. 1 Suregrow, Brogaarden Diets Ltd. Lynge, Denmark

Analytical Constituents:

Crude Protein	0,25%
Crude Oil and fats	5.4%
Crude Fiber	8.5%
Crude Ash	0,12%
Sodium	0.5%

Digestible Energy MJ/kg: 11.75

Calcium	2.5%	Vitamin K	6mg/kg
Phosphorus	1.5%	Biotin	4.25mg/kg
Magnesium	0.4%	Pantothenic acid	65mg/kg
Salt	1.4%	Nicotinic acid	90mg/kg
Sodium	0.5%	Folic acid	80mg/kg
Potassium	1.2%	Choline Chloride	2000mg/kg
Sulphur	0.23%	Cobalt	1.1mg/kg
Lysine	18g/kg	Copper	165mg/kg
Methionine	3.7g/kg	Iron	250mg/kg
Vitamin A	54000Iu/kg	Manganese	350mg/kg
Vitamin D_3	5400iu/kg	Selenium	1.9mg/kg
Vitamin E	1200mg/kg	Zinc	550mg/kg
Vitamin B_1	62mg/kg	Iodine	2.2mg/kg
Vitamin B_2	25mg/kg	Starch	12-14%
Vitamin B_6	44mg/kg	Sugar	5-6%
Vitamin B_{12}	0.09mg/kg	DCAB	315mequiv
NDF	0,22	ADF	0,1

Brogaarden Lucerne Mix, Brogaarden Diets Ltd. Lynge, Denmark

Analytical Constituents:	
Crude Protein	13.0%
Crude Oil and fats	2.5%
Crude Fiber	31.0%
Crude Ash	10.0%
Digestible Energy	
MJ/kg: 7.0	
Calcium	1.2%
Phosphorus	0.3%
Magnesium	0.15%
Sodium	0.08%
Lysine	0.53%

Supplemental table 1: Dietary composition
