# Supplementary material for: An Oligosaccharide Rich Diet Increases Akkermansia spp. Bacteria in the Equine Microbiota
Source: Front Microbiol. 2021 May 21;12:666039. doi: 10.3389/fmicb.2021.666039 (PMC8176217; doi:10.3389/fmicb.2021.666039)
Supplement: Supplementary file 2 [file Data_Sheet_2.docx]

| **Study** | **Pretreatment** | **Laminitis induction dose** |
| --- | --- | --- |
| Tadros, Frank et al. 2012 ^1^ |  | 5 g/kg |
| van Eps, Leise et al. 2012 ^2^ |  | 10 g/kg |
| Visser, Pollitt 2012 ^3^ |  | 10 g/kg |
| Visser, Pollitt 2011 ^4^ |  | 10 g/kg |
| (Keller, Pollitt et al. 2011 ^5^ |  | 7,5 g/kg |
| de Laat, van Eps et al. 2011 ^6^ |  | 10 g/kg |
| (Uberti, Pressler et al. 2010 ^7^ | 3x1 g/kg with 24h interval | 10 g/kg |
| Milinovich, Klieve et al. 2010 ^8^ |  | 7,5 g/kg |
|  |  | 10 g/kg |
|  |  | 12,5 g/kg |
| Budak, Orsini et al. 2009 ^9^ |  | 10 g/kg |
| Toth, Frank et al. 2009 ^10^ |  | 5 g/kg |
| Kalck, Frank et al. 2009 ^11^ | 1 g/kg daily in 6 days | 5 g/kg |
|  |  | 7,5 g/kg |
| Van Eps, Pollitt 2009 ^12^ |  | 10 g/kg |
| Bailey, Adair et al. 2009 ^13^ |  | 10 g/kg |
| Milinovich, Burrell et al. 2008 ^14^ |  | 10 g/kg |
| Milinovich, Burrell et al. 2008 ^15^ |  | 10 g/kg |
| Nourian, Baldwin et al. 2007 ^16^ |  | 10 g/kg |
| Milinovich, Trott et al. 2007 ^17^ |  | 10 g/kg |
| van Eps, Pollitt 2006 ^18^ |  | 7,5 g/kg |
|  |  | 10 g/kg |
|  |  | 12,5 g/kg |
| Milinovich, Trott et al. 2006 ^19^ |  | 10 g/kg |
| French, Pollitt 2004 ^20^ |  | 7,5 g/kg |
|  |  | 10 g/kg |
|  |  | 12,5 g/kg |

| **Study** | **Dose** | **FOS as Prebiotics** |
| --- | --- | --- |
| Respondek, Myers et at. 2010^21^ |  | 0,08 g/kg |
| Respondek, Goachet et al. 2007^22^ |  | 0,01 g/kg |
| Berg, Fu et al. 2005^23^ | Low dose | 0,0199 g/kg |
|  | High dose | 0,0599 g/kg |
| 1. Tadros EM, Frank N, Newkirk KM, Donnell RL, Horohov DW. Effects of a "two-hit" model of organ damage on the systemic inflammatory response and development of laminitis in horses. *Vet Immunol Immunopathol*. 2012;150(1-2):90-100.  2. van Eps AW, Leise BS, Watts M, Pollitt CC, Belknap JK. Digital hypothermia inhibits early lamellar inflammatory signalling in the oligofructose laminitis model. *Equine Vet J*. 2012;44(2):230-237.  3. Visser MB, Pollitt CC. The timeline of metalloprotease events during oligofructose induced equine laminitis development. *Equine Vet J*. 2012;44(1):88-93.  4. Visser MB, Pollitt CC. Lamellar leukocyte infiltration and involvement of IL-6 during oligofructose-induced equine laminitis development. *Vet Immunol Immunopathol*. 2011;144(1-2):120-128.  5. Keller MD, Pollitt CC, Marx UC. Nuclear magnetic resonance-based metabonomic study of early time point laminitis in an oligofructose-overload model. *Equine Vet J*. 2011;43(6):737-743.  6. de Laat MA, van Eps AW, McGowan CM, Sillence MN, Pollitt CC. Equine laminitis: Comparative histopathology 48 hours after experimental induction with insulin or alimentary oligofructose in standardbred horses. *J Comp Pathol*. 2011;145(4):399-409.  7. Uberti B, Pressler BM, Alkabes SB, et al. Effect of heparin administration on urine protein excretion during the developmental stage of experimentally induced laminitis in horses. *Am J Vet Res*. 2010;71(12):1462-1467.  8. Milinovich GJ, Klieve AV, Pollitt CC, Trott DJ. Microbial events in the hindgut during carbohydrate-induced equine laminitis. *Vet Clin North Am Equine Pract*. 2010;26(1):79-94.  9. Budak MT, Orsini JA, Pollitt CC, Rubinstein NA. Gene expression in the lamellar dermis-epidermis during the developmental phase of carbohydrate overload-induced laminitis in the horse. *Vet Immunol Immunopathol*. 2009;131(1-2):86-96.  10. Toth F, Frank N, Chameroy KA, Bostont RC. Effects of endotoxaemia and carbohydrate overload on glucose and insulin dynamics and the development of laminitis in horses. *Equine Vet J*. 2009;41(9):852-858.  11. Kalck KA, Frank N, Elliott SB, Boston RC. Effects of low-dose oligofructose treatment administered via nasogastric intubation on induction of laminitis and associated alterations in glucose and insulin dynamics in horses. *Am J Vet Res*. 2009;70(5):624-632.  12. Van Eps AW, Pollitt CC. Equine laminitis model: Cryotherapy reduces the severity of lesions evaluated seven days after induction with oligofructose. *Equine Vet J*. 2009;41(8):741-746.  13. Bailey SR, Adair HS, Reinemeyer CR, et al. Plasma concentrations of endotoxin and platelet activation in the developmental stage of oligofructose-induced laminitis. *Vet Immunol Immunopathol*. 2009;129(3-4):167-173.  14. Milinovich GJ, Burrell PC, Pollitt CC, et al. Microbial ecology of the equine hindgut during oligofructose-induced laminitis. *ISME J*. 2008;2(11):1089-1100.  15. Milinovich GJ, Burrell PC, Pollitt CC, Bouvet A, Trott DJ. Streptococcus henryi sp. nov. and streptococcus caballi sp. nov., isolated from the hindgut of horses with oligofructose-induced laminitis. *Int J Syst Evol Microbiol*. 2008;58(Pt 1):262-266.  16. Nourian AR, Baldwin GI, van Eps AW, Pollitt CC. Equine laminitis: Ultrastructural lesions detected 24-30 hours after induction with oligofructose. *Equine Vet J*. 2007;39(4):360-364.  17. Milinovich GJ, Trott DJ, Burrell PC, et al. Fluorescence in situ hybridization analysis of hindgut bacteria associated with the development of equine laminitis. *Environ Microbiol*. 2007;9(8):2090-2100.  18. van Eps AW, Pollitt CC. Equine laminitis induced with oligofructose. *Equine Vet J*. 2006;38(3):203-208.  19. Milinovich GJ, Trott DJ, Burrell PC, et al. Changes in equine hindgut bacterial populations during oligofructose-induced laminitis. *Environ Microbiol*. 2006;8(5):885-898.  20. French KR, Pollitt CC. Equine laminitis: Loss of hemidesmosomes in hoof secondary epidermal lamellae correlates to dose in an oligofructose induction model: An ultrastructural study. *Equine Vet J*. 2004;36(3):230-235.  21. Respondek F, Myers K, Smith TL, Wagner A, Geor RJ. Dietary supplementation with short-chain fructo-oligosaccharides improves insulin sensitivity in obese horses. *J Anim Sci*. 2011;89(1):77-83.  22. Respondek, F. Goachet, A. G. Julliand, Julliard. Effects of dietary short-chain fructo-oligosaccharides on the intestinal microflora of horses subjected to a sudden change in diet. *Journal of animal science*. 2007;October 16.  23. Berg EL, Fu CJ, Porter JH, Kerley MS. Fructooligosaccharide supplementation in the yearling horse: Effects on fecal pH, microbial content, and volatile fatty acid concentrations. *J Anim Sci*. 2005;83(7):1549-1553. | | |

Supplementary Table 2:

Overview of doses of fructooligosaccharides (FOS) in studies studies using FOS for laminitis induction and as a prebiotic respectively
